# Machine learning algorithms for systematic review: reducing workload in a preclinical review of animal studies and reducing human screening error

**DOI:** 10.1186/s13643-019-0942-7

**Published:** 2019-01-15

**Authors:** Alexandra Bannach-Brown, Piotr Przybyła, James Thomas, Andrew S. C. Rice, Sophia Ananiadou, Jing Liao, Malcolm Robert Macleod

**Affiliations:** 1 Centre for Clinical Brain Sciences, University of Edinburgh, Edinburgh, Scotland; 2 National Centre for Text Mining, School of Computer Science, University of Manchester, Manchester, England; 3 EPPI-Centre, Department of Social Science, University College London, London, England; 4 Pain Research, Department of Surgery and Cancer, Imperial College, London, England; 5 Translational Neuropsychiatry Unit, Aarhus University, Aarhus, Denmark; 6 Present Address: Centre for Research in Evidence-Based Practice, Bond University, Gold Coast, Australia

**Keywords:** Machine learning, Systematic review, Analysis of human error, Citation screening, Automation tools

## Abstract

**Background:**

Here, we outline a method of applying existing machine learning (ML) approaches to aid citation screening in an on-going broad and shallow systematic review of preclinical animal studies. The aim is to achieve a high-performing algorithm comparable to human screening that can reduce human resources required for carrying out this step of a systematic review.

**Methods:**

We applied ML approaches to a broad systematic review of animal models of depression at the citation screening stage. We tested two independently developed ML approaches which used different classification models and feature sets. We recorded the performance of the ML approaches on an unseen validation set of papers using sensitivity, specificity and accuracy. We aimed to achieve 95% sensitivity and to maximise specificity. The classification model providing the most accurate predictions was applied to the remaining unseen records in the dataset and will be used in the next stage of the preclinical biomedical sciences systematic review. We used a cross-validation technique to assign ML inclusion likelihood scores to the human screened records, to identify potential errors made during the human screening process (error analysis).

**Results:**

ML approaches reached 98.7% sensitivity based on learning from a training set of 5749 records, with an inclusion prevalence of 13.2%. The highest level of specificity reached was 86%. Performance was assessed on an independent validation dataset. Human errors in the training and validation sets were successfully identified using the assigned inclusion likelihood from the ML model to highlight discrepancies. Training the ML algorithm on the corrected dataset improved the specificity of the algorithm without compromising sensitivity. Error analysis correction leads to a 3% improvement in sensitivity and specificity, which increases precision and accuracy of the ML algorithm.

**Conclusions:**

This work has confirmed the performance and application of ML algorithms for screening in systematic reviews of preclinical animal studies. It has highlighted the novel use of ML algorithms to identify human error. This needs to be confirmed in other reviews with different inclusion prevalence levels, but represents a promising approach to integrating human decisions and automation in systematic review methodology.

**Electronic supplementary material:**

The online version of this article (10.1186/s13643-019-0942-7) contains supplementary material, which is available to authorized users.

## Background

The rate of publication of primary research is increasing exponentially within biomedicine [1]. Researchers find it increasingly difficult to keep up with new findings and discoveries even within a single biomedical domain, an issue that has been emerging for a number of years [2]. Synthesising research—either informally or through systematic reviews—becomes increasingly resource intensive, as searches retrieve larger and larger corpora of potentially relevant papers for reviewers to screen for relevance to the research question at hand.

This increase in rate of publication is seen in the animal literature. In an update to a systematic review of animal models of neuropathic pain, 11,880 further unique records were retrieved in 2015, to add to 33,184 unique records identified in a search conducted in 2012. In the field of animal models of depression, the number of unique records retrieved from a systematic search increased from 70,365 in May 2016 to 76,679 in August 2017.

The use of text-mining tools and machine learning (ML) algorithms to aid systematic review is becoming an increasingly popular approach to reduce human burden and monetary resources required and to reduce the time taken to complete such reviews [3–5]. ML algorithms are primarily employed at the screening stage in the systematic review process. This screening stage involves categorising records identified from the search into ‘relevant’ or ‘not-relevant’ to the research question, typically performed by two independent human reviewers with discrepancies reconciled by a third. This decision is typically made on the basis of the title and abstract of an article in the first instance. In previous experience at CAMARADES (Collaborative Approach to Meta-Analysis and Review of Animal Data from Experimental Studies), screening a preclinical systematic review with 33,184 unique search results took 9 months, representing (because of dual screening) around 18 person-months in total. Based partly on this, we estimate that a systematic review with roughly 10,000 publications retrieved takes a minimum of 40 weeks. In clinical systematic reviews, Borah and colleagues [6] showed the average clinical systematic review registered on PROSPERO (International Prospective Register of Systematic Reviews) takes an average 67.3 weeks to complete. ML algorithms can be employed to learn this categorisation ability, based on training instances that have been screened by human reviewers [7].

Several applications of ML are possible. The least burdensome is when a review is being updated, where categorisations from the original review are used to train a classifier, which is then applied to new documents identified in the updated search [7–9]. When a screening is performed de novo, without such previous collection, humans first categorise an initial set of search returns, which are used to train an ML model. The performance of the model is then tested (either in a validation set or with k fold cross validation); if performance does not meet a required threshold then more records are screened, chosen either through random sampling or, using active learning [10], on the basis either of those with highest uncertainty of predictions [11, 12] or alternatively from those most likely to be included [13–15]. Here, we use a de novo search with subsequent training sets identified by random sampling, and we introduce a novel use of machine prediction in identifying human error in screening decisions.

Machine learning approaches have been evaluated in context of systematic reviews of several medical problems including drug class efficacy assessment [7, 8, 12], genetic associations [9], public health [13, 16], cost-effectiveness analyses [9], toxicology [3], treatment effectiveness [17, 18], and nutrition [17]. To the best of our knowledge, there have been only two attempts to apply such techniques to reviews of preclinical animal studies [3, 19]. These can be broad and shallow reviews or focussed and detailed reviews and can have varying prevalence of inclusion.

Here, we outline the ML approach taken to assist in screening a corpus for a broad and shallow systematic review seeking to summarise studies using non-human animal models of depression, based on a corpus of 70,365 records retrieved from two online biomedical databases. In this paper, our aim was to train an algorithm to achieve the level of performance of two independent human screeners, so that we might reduce the human resource required.

Sena and colleagues developed guidelines for the appraisal of systematic reviews of animal studies [20]. These guidelines consider dual extraction by two independent human reviewers as a feature of a high-quality review. From a large corpus of reviews conducted by CAMARADES (Collaborative Approach to Meta-Analysis and Review of Animal Data from Experimental Studies), we estimate the inter-screener agreement to be between 95% and 99%. Errors may occur at random (due to fatigue or distraction) or, more consequentially, systematic human biases or errors, which, if included in a training set, might be propagated into a ML algorithm. Certain types of records might be at greater risk of misclassification if systematic errors are present. To our knowledge, the nature of this 5% residual human error in systematic review methodology has not been formally investigated. The training data used for ML categorisation is based on training instances that has been screened by two independent human screeners. Each record is presented to any given reviewer at random to reduce any effects of screening records in a specific order.

We therefore aimed to explore the use of established ML algorithms as part of the classification stage in a preclinical, to investigate if the ML algorithms could be used to improve the human gold standard by identifying human screening errors and thus improve the overall performance of ML.

## Methods

**Fig. 1:**
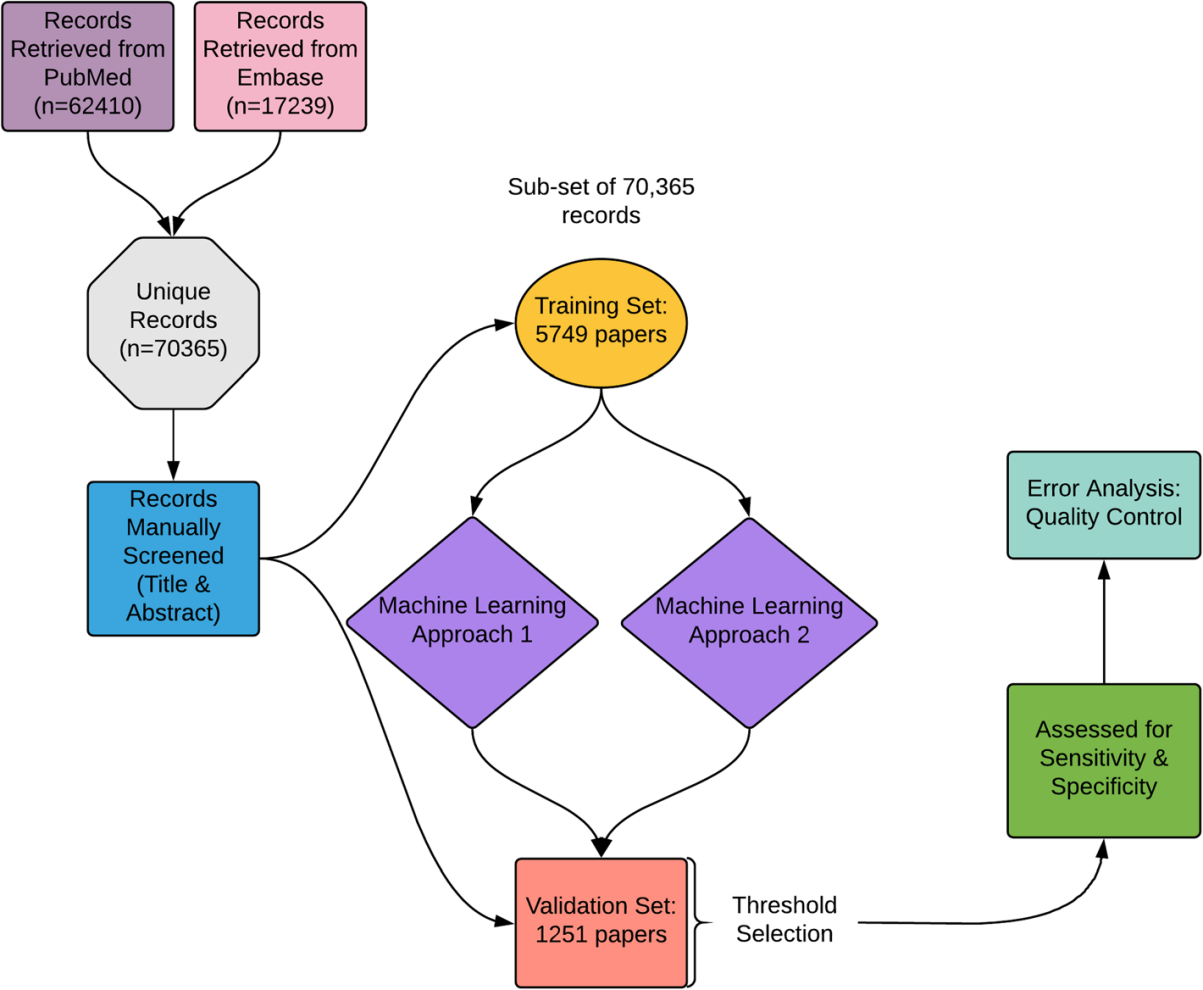
Diagram of the layout of the study

We applied two independent machine learning approaches to screening a large number of identified citations (70,365 records) for a systematic review. We first selected 2000 records at random to provide the first training set. This number was chosen arbitrarily as we could not predict how many training instances would be required. Of these, only 1993 were suitable due to data deposition errors. These were then screened by two human reviewers with previous experience with reviews of animal studies, with a third expert reviewer reconciling any differences. The resulting ML algorithms gave a score between 0 and 1. To ensure that the true sensitivity was likely to be 95% or higher, we chose as our cut-point the value for which the lower bound of the 95% confidence interval of the observed sensitivity exceeded 95% when applied to the unseen validation dataset. We then repeated this process adding a further 1000 randomly selected (996 useable) citations to the training set; and then again adding a further 3000 randomly selected (2760 useable) citations to the training set. At each stage, we assessed performance of the approaches on a validation set of unseen documents, using a number of different metrics. Next, the best performing algorithm was used to identify human errors in the training and validation sets by selecting those with the largest discrepancy between the human decision (characterised as 0 for exclude or 1 for include) and the machine prediction (a continuous variable between 0 and 1). Performance of the approaches trained on the full 5749 records is reported here, and each of the iterations is available in Additional file 1. The error analysis was assessed on the net reclassification index, and the performance of the ML approach is compared before and after correcting the errors in human screening using AUC (Fig. 1).

### Step 1: Application of ML tools to screening of a large preclinical systematic review

#### Training sets

We identified 70,365 potentially relevant records from PubMed and EMBASE. The search strings were composed of the animal filters devised by the Systematic Review Center for Laboratory animal Experimentation (SYRCLE) [21, 22], NOT reviews, comments, or letters AND a depression disorder string (for full search strings see [23]). The training and validation sets were chosen at random from the 70,365 by assigning each record a random number using the RAND function in excel and ranking them from smallest to largest. The final training set consisted of 5749 records. The final validation set consisted of the next 1251 records. The training set and validation sets were screened by two independent human screeners with any discrepancies reconciled by a third independent human screener. The human screening process used an online systematic review tool called SyRF (app.syrf.org) which randomly presents a reviewer with a record, with the title and abstract displayed. The reviewer makes a decision about the record, to include (1) or to exclude (0). A second reviewer is also presented with records but in a different random sequence. If a given record receives two ‘include’ decisions or two ‘exclude’ decisions, the screening for this record is considered complete. If reviewer 1 and reviewer 2 disagree, the record is listed for review by third reviewer who. The record then has an average inclusion score of 0.666 or 0.333. Any record that has an inclusion score above 0.6 is included, those scoring less than 0.6 are excluded, and screening is considered complete. Reviewers are not aware of whether they are the first, second or third reviewer or of the decisions of the other reviewers. Datasets are available on Zenodo, as described in the “Availability of data and materials” section. The validation set had more than 150 ‘included’ records, which should give a reasonably precise estimate of the sensitivity and specificity which would be achieved in screening other citations from the population from which the validation set was drawn.

Three feature sets (BoW, LDA and SVD (LSI)) were tested on SVMs, logistic regression and random forests [24]. The two algorithms described below performed best for this dataset of 70,365 records, on the broad topic of preclinical animal models of depression.

#### Approaches

Here, two approaches were developed independently, using different classification models and feature representations, but sharing the linear classification principles.

##### Approach 1

Approach 1 used a tri-gram ‘bag-of-words’ model for feature selection and implemented a linear support vector machine (SVM) with stochastic gradient descent (SGD) as supported by the SciKit-Learn python library [25]. To account for the relative importance of words within a given document, and difference in words used between documents we used ‘Term Frequency – Inverse Document Frequency’ (TD-IDF). This is defined as

$$ tfidf\left({w}_i,{d}_j\right)= tf\left({w}_i,{d}_j\right)\ast \frac{\left|D\right|}{\left|\left\{d:{w}_i\in d\right\}\right|} $$

The score for the *i*th word in context of the *j*th document takes into account not only how many times the word occurred there (tf), but also how many other documents (*d*) from the whole corpus (*D*) contain it as well. This helps to reduce the score for words that are common for all documents and therefore have little predictive power. This helps the classifier to focus on terms which help to distinguish between documents, rather than on terms which occur frequently [26]. We allowed n-grams; did not use stemming; and used the MySQL text indexing functionality ‘stopword’ list to remove frequently occurring words which provide little relevant information for classification purposes [27].

The support vector machine classifier with stochastic gradient descent (SGD) was chosen as it is efficient, scales well to large numbers of records, and provides an easily interpretable list of probability estimates when predicting class membership (i.e. scores for each document lying between 0 and 1). Efficiency and interpretability are important, as this classifier is already deployed in a large systematic review platform [28], and any deployed algorithm therefore needs not to be too computationally demanding, and its results understood by users who are not machine learning specialists. The tri-gram feature selection approach without any additional feature engineering also reflects the generalist need of deployment on a platform used in a wide range of reviews: the algorithm needs to be generalisable across disciplines and literatures, and not ‘over-fitted’ to a specific area. For example, the tri-gram ‘randomised controlled trial’ has quite different implications for classification compared with ‘randomised controlled trials’ (i.e. ‘trials’ in plural). The former might be a report of a randomised controlled trial; while the latter is often found in reports of systematic reviews of randomised trials. Stemming would remove the ‘s’ on trials and thus lose this important information. This approach aims to give the best compromise between reliable performance across a wide range of domains and that achievable from a workflow that has been highly tuned to a specific context.

##### Approach 2

Approach 2 used a regularised logistic regression model built on latent Dirichlet allocation (LDA) and singular value decomposition (SVD) features. Namely, the document text (consisting of title and abstract) was first lemmatised with the tool GENIA tagger [29] and then converted into bag-of-words representation of unigrams, which was then used to create two types of features. First, the word frequencies were converted into a matrix TF/IDF scores, which was then decomposed via a general matrix factorisation technique (SVD) implemented in scikit-learn library and truncated to the first 300 dimensions. Second, an LDA model was built using MALLET library [30], setting 300 as a number of topics. As a result, each document was represented by 600 features, and an L1-regularised logistic regression model was built using glmnet package [31] in R statistical framework [32].

In this procedure, every document is represented with a constant, manageable number of features, irrespective of corpus or vocabulary size. As a result, we can use a relatively simple classification algorithm and expect good performance with short processing time even for very large collections. This feature is particularly useful when running the procedure numerous times in cross-validation mode for error analysis (see below).

For further details of feature generation methods and classifiers see Additional file 1. For a given unseen test instance, the logistic regression returns a score corresponding to the probability of it being relevant according to the current model. An optimal cut-off score that gives the best performance is calculated as described above.

#### Assessing machine learning performance

The facets of a machine learning algorithm performance that would be most beneficial to this field of research are high sensitivity (see Table 1), at a level comparable to the 95% we estimate is achieved by two independent human screeners. To be confident that the sensitivity which would be achieved in the screening of other publications from which the validation set was drawn would be 95% or higher, we selected the threshold for inclusion such that the lower bound of the 95% confidence interval of the observed sensitivity in the validation set excluded 95% sensitivity. This has practical implications that, the larger the validation set, the more precisely that sensitivity will be estimated. Once the level of sensitivity has been reached, the next priority is to maximise specificity, to reduce the number of irrelevant records included by an algorithm. Although specificity at 95% sensitivity is our goal, we also provide additional measures of performance.

**Table 1:** Equations used to assess performance of machine learning algorithms

Sensitivity or recall	TP/(TP + FN)
Specificity	TN/(TN + FP)
Precision	TP/(TP + FP)
Accuracy	(TP + TN)/(TP + FP + FN + TN)
WSS@95%	((TN + FN)/N) – (1.0 – 0.95)
Positive likelihood ratio (LR+)	(Sensitivity)/(1-specificity)

Sensitivity, specificity, precision, accuracy and WSS@95% equations from [5]. Positive likelihood ratio equation from [45]

##### Performance metrics

Performance was assessed using sensitivity (or recall), specificity, precision, accuracy, work saved over sampling (WSS), and the positive likelihood ration (LR+) (see Table 1), carried out in R (R version 3.4.2; [32]) using the ‘caret’ package [24]. 95% confidence intervals were calculated using the efficient-score method [33]. Cut-offs were determined manually for each approach by taking the score that gave confidence that true sensitivity was at least 95% (as described above), and the specificity at this score was calculated.

### Step 2: Application of ML tools to training datasets to identify human error

#### Error analysis methods

**Fig. 2:**
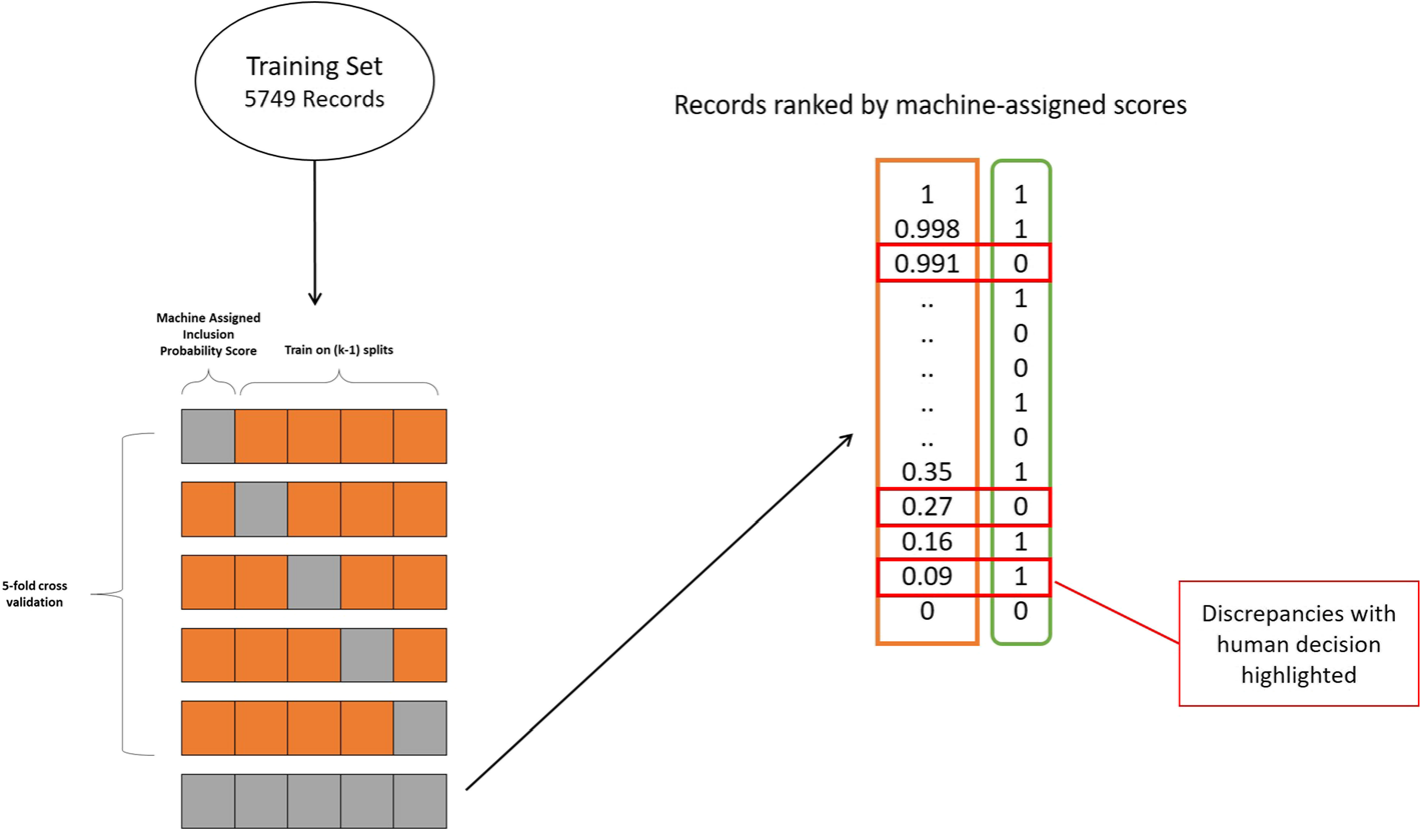
Error analysis. The methodology for using cross-validation to assign ML-predicted probability scores. The ML-predicted probability scores for the records were checked against the original human inclusion decision

The approach to error analysis was outlined in an a priori protocol, published on the CAMARADES (Collaborative Approach to Meta-Analysis and Review of Animal Data from Experimental Studies) website on 18 December 2016 [34]. We used non-exhaustive fivefold cross-validation to generate the machine learning scores for the set of records that were originally used to train the machine (5749 records). This involves randomly partitioning records into five equal sized subsamples. Over five iterations, one subsample is set aside, and the remaining four subsamples are used to train the algorithm [35]. Thus, every record serves as an ‘unknown’ in one of these iterations, and has a score computed by a machine learning model where it was not included in the training portion. These scores were used to highlight discrepancies or disagreements between machine decision and human decision. The documents were ranked by the machine assigned prediction of relevance from most likely to least likely. The original human assigned scores (either 0 or 1) were compared with this ranking, to highlight potential errors in the human decision. A single human reviewer (experienced in animal systematic reviews) manually reassessed the records starting with the most discrepant. To avoid reassessing the full 5749 record dataset, a pragmatic stopping rule was established such that if the initial human decision was correct for five consecutive records, further records were not reassessed (Fig. 2).

After the errors in the training set were investigated and corrected as described above, a second model was built on the updated training data. The outcome of error analysis is presented as reclassification tables, the area under the curve (AUC) being used to compare the performance of the ML algorithm trained on the uncorrected training set, and the net reclassification index (NRI) [36] used to compare the performance of the classifier built on the updated training data with the performance of the classifier built on the original training data. The following equation was used [37]:

$$ {\mathrm{NRI}}_{\mathrm{binary}\ \mathrm{outcomes}}={\left(\mathrm{Sensitivity}+\mathrm{Specificity}\right)}_{\mathrm{second}\ \mathrm{test}}-{\left(\mathrm{Sensitivity}+\mathrm{Specificity}\right)}_{\mathrm{first}\ \mathrm{test}} $$

The AUC was calculated using the DeLong method in the ‘pROC’ package in R [38].

Further, we applied the same technique as above to identify human screening errors in the validation dataset. Due to the small number of records in the validation set (1251 records), it was assumed that every error would be likely to impact measured performance, and so the manual screening of the validation set involved revisiting every record where the human and machine decision were incongruent. The number of reclassified records was noted. The inter-rater reliability of all screening decisions on training set and validation set between reviewer 1 and reviewer 2 were analysed using the ‘Kappa.test’ function in the ‘fmsb’ package in R [39].

## Results

We first describe the performance from the ML algorithms, then show the results from the analysis of human error, and finally describe the performance of the ML algorithm after human errors in the training and validation set have been corrected.

### Performance of machine learning algorithms

**Fig. 3:**
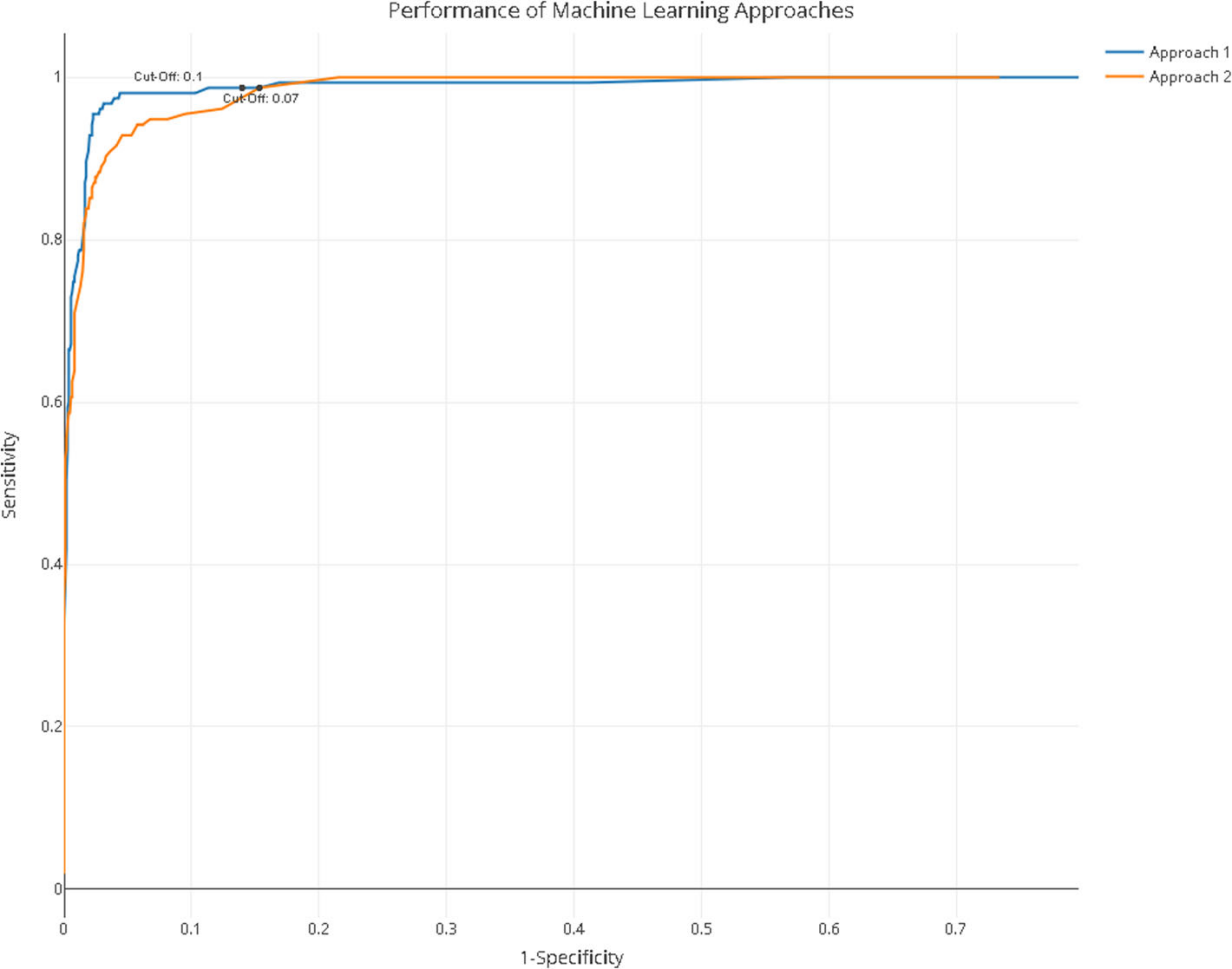
Performance of machine learning approaches. For the interactive version of this plot with cut-off values, see code and data at https://github.com/abannachbrown/The-use-of-text-mining-and-machine-learning-algorithms-in-systematic-reviews/blob/master/ML-fig3.html

Table 2 shows the performance of the two machine learning approaches from the SLIM (Systematic Living Information Machine) collaboration. The desired sensitivity of 95% (including lower bound 95% CI) has been reached by both approaches. Both approaches reached 98.7% sensitivity based on learning from a training set of 5749 records, with an inclusion prevalence of 13.2% (see below). Approach 1 reached a higher specificity level of 86%. This is visualised on an AUC curve (Figs. 1 and 3).

**Table 2:** Performance of machine learning approaches on depression training dataset

	Approach 1	Approach 2
Training set size	5749	5749
Optimal cut-off score	0.1	0.07
Sensitivity	98.7%	98.7%
Upper 95% CI	0.997	0.997
Lower 95% CI	0.949	0.949
Specificity	86.0%	84.7%
Precision	50%	47.66%
Accuracy	1096/1251 = 87.6%	1081/1251 = 86.4%
WSS@95%	0.705	0.693
LR+	7.421	9.451

### Error analysis and reclassification

Interrater agreement (Cohen’s κ) between the screening decisions of reviewer 1 and reviewer 2 was 0.791 (95% CI, 0.769 to 0.811, *p* < 0.0001), with 281 records requiring a third reviewer decision. To assess whether machine learning algorithms can identify human error and therefore improve the training data, we conducted an error analysis. We reassessed papers where the ML predictive scores were highly divergent from human assigned labels to identify potential human errors. After the 75 most divergent papers had been rescreened, the machine corrected the human decision 47 times and the initial human decision was correct 28 times. We also rescreened the validation set. Ten papers out of the 1251 records were highly divergent and identified as potential human errors. Of these, the machine corrected eight human decisions where the record had been wrongly excluded; the initial human decision was correct twice (see the “Error analysis methods” section for details on error determination process).

The identified human error in the training set was 47of 5749 records, or 0.8%, and this is therefore the lowest possible error of the reconciled human decisions; the true error is likely to be higher. Of the 47, 11 records had been wrongly included and 36 records had been wrongly excluded. We consider wrongful exclusion of relevant records as more troublesome than wrongful inclusion (hence our emphasis on sensitivity over specificity), and the application of the error correction approach increased the number of correctly included studies from 759 in the reconciled human screen (760 less the 11 wrongly included studies) to 795, an increase of 4.7%.

Similarly, the human error rate in the validation set (1251 records) was 0.6%. Considering the prevalence of inclusion in this dataset (155/1251, 12.4%) rising to 163/1251, 13.0%), the 8 reclassified records represent a 4.9% increase in the number of correctly included studies.

- Test 1: 98.7% + 86% = 184.7%
- Test 2: 98.2% + 89.3% = 187.5%
- NRI = 3.2%

We consider the updated validation set as the revised gold standard. The confusion matrix for the performance of the machine learning algorithm after the error analysis update on the training records is shown in Table 3.

**Table 3:** Reclassification of records in validation after error analysis

	Test 1—original machine learning algorithms results			
		In	Out	Total
Test 2—post-error analysis ML results	In	153	153	306
		160	116	276
	Out	2	943	945
		3	972	975
	Total	155	1096	1251
		163	1088	

Analysing the human errors identified by the machine learning algorithm and correcting for these errors and re-teaching the algorithm leads to improved performance of the algorithm, particularly its sensitivity. Analysing human errors can save considerable human time in the screening stage of a systematic review. Consider the remaining approximately 64,000 papers, if the ML algorithm results are 3% more accurate, that is approximately 2000 papers that are correctly ‘excluded’ that would not be forwarded for data extraction.

### After error analysis: improving machine learning

**Fig. 4:**
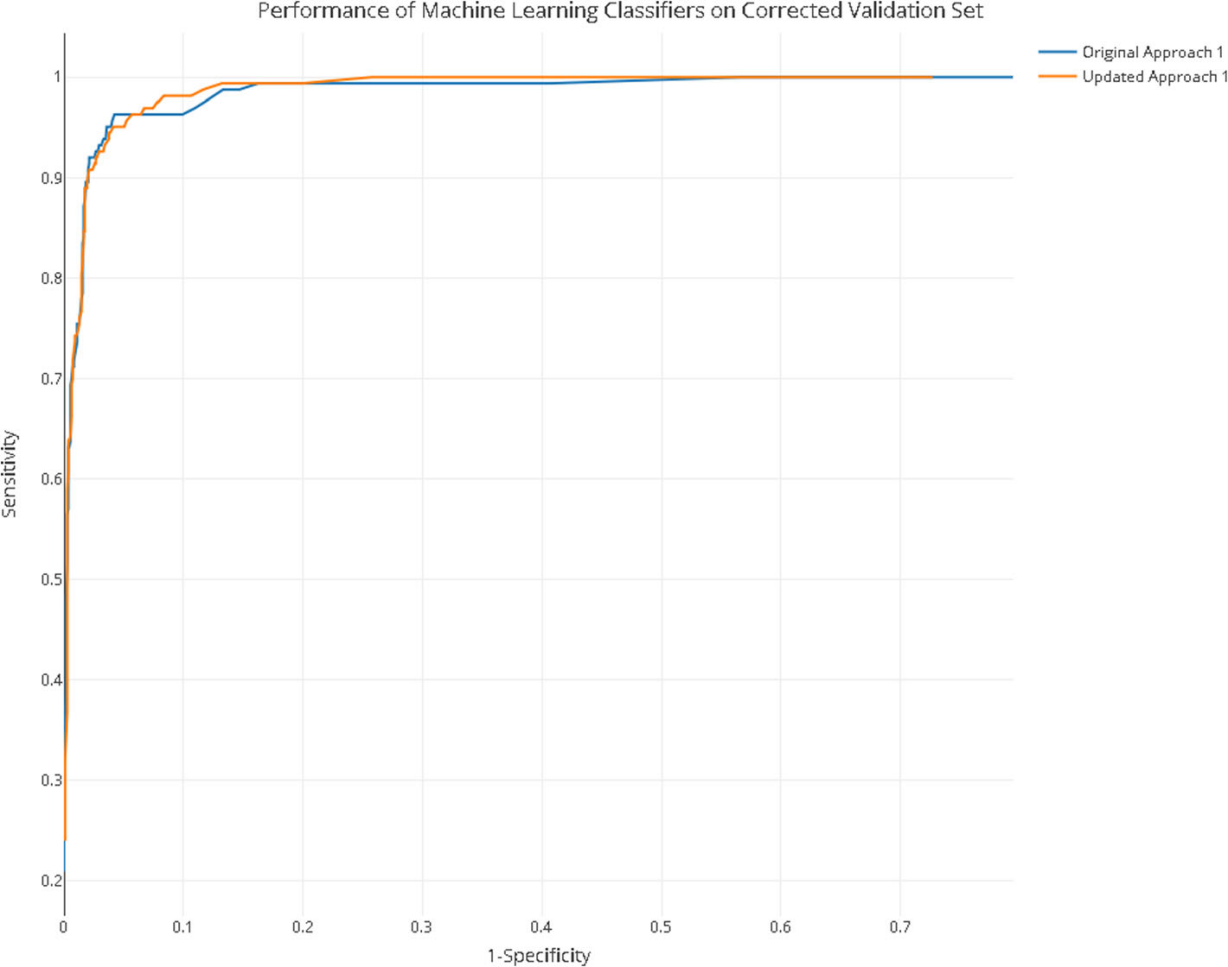
Performance of approach 1 after error analysis. The updated approach is retrained on the corrected training set after error analysis correction. Performance on both the original and the updated approach is measured on the corrected validation set (with error analysis correction). For the interactive version of this plot with the ability to read off performance at all cut-off values, see code and data at https://github.com/abannachbrown/The-use-of-text-mining-and-machine-learning-algorithms-in-systematic-reviews/blob/master/error-analysis-plot.html

Using the error analysis technique above, of the 47 errors identified in the full training dataset of 5749 records, 0.8% were corrected. We retrained approach 1 on the corrected training set and measured performance on the corrected validation set of 1251 records, the revised gold standard. The performance of the original approach 1 and updated approach 1 was assessed on the corrected validation set of 1251 records. The performance of this retrained algorithm in comparison to the performance of the original classifier 5 on the updated validation set is shown in Table 4 (Fig. 4).

**Table 4:** Performance of machine learning approach after error analysis

	Updated approach 1	Original approach 1
Cut-off	0.09	0.10
Sensitivity	98.7%	98.7%
Upper 95% CI of sensitivity	0.997	0.997
Lower 95% CI of sensitivity	0.949	0.949
Specificity	88.3%	86.7%
Precision	55.9%	52.61%
Accuracy	89.7%	88.2%
WSS@95%	961/1251 – (0.05) = 0.718	945/1251 – (0.05) = 0.705
LR+	8.436	7.421

We compared the area under the ROC curve for the original and the updated approach. The AUC increased from 0.927 (95% CI (DeLong); 0.914–0.9404) for the original to 0.935 (95% CI 0.9227–0.9483) for the updated approach (DeLong’s test of difference in AUC *Z* = − 2.3685, *p* = 0.018).

## Discussion

### Document classification

We have shown machine learning algorithms to have high levels of performance for ascertainment of relevant publications describing animal experiments modelling depression, with 98.7% sensitivity and 88.3% specificity. This is comparable to the performance of dual human screening with reconciliation. The objectives for selecting ML approaches in this project was to be confident that the sensitivity achieved in citations drawn from the same population as the validation set would be at least 95%. Thereafter, algorithms were then chosen based on their specificity; our workflow would have all papers included at the stage progress to full-text retrieval, annotation and data extraction, and we wish to minimise unnecessary labour at this stage. We recognise that the estimated precision (55.9%) is low. However, reviews of the animal literature often seek to summarise all information relevant to the modelling of a particular disease or to a category of intervention, and in this context the absolute benefits can be transformative, allowing reviews to be conducted which would not otherwise be feasible. In this example, the number of documents needing to be screened by humans is reduced from over 70,000 to only 18,500 documents; even if half of those are falsely included to the full-text annotation stage, there is still a saving in screening of 50,000 documents, at least 100,000 screening events, representing several months of investigator effort.

The precision estimate achieved by this classifier will result in different performance in datasets with different inclusion prevalence. To guide potential users, we have simulated the likely achieved precision when applying the best performing machine learning here to projects with different inclusion prevalence (Additional file 2: Figure S1, Additional file 3: Data S1).

The two machine learning approaches have similar performance, and the slight differences observed may reflect the method of feature generation. These algorithms both have high performance on this specific topic of animal models of depression. As demonstrated previously, the performance of various classifiers can alter depending on the topic and specificity of the research question [3].

In this study, the cut-off points were selected using the decisions on the validation set to achieve the desired performance. Although this allows the measurement of the maximum possible gain using a given approach in an evaluation setting, in practice (e.g. when updating a review), the true scores would not be available. The problem of choosing a cut-off threshold, equivalent to deciding when to stop when using a model for prioritising relevant documents, remains an open research question in information retrieval. Various approaches have been tested [40], but they do not guarantee achieving a desired sensitivity level. Our preferred approach is to use the threshold identified in a validation set and to apply it to the remaining ‘unknown’ records. ML-based approaches can also be used without a cut-off where all documents are screened manually, but those most likely to be included are screened first to optimise workflows thus reducing the workload [5]. In a similar broad preclinical research project in neuropathic pain, it took 18 person-months to screen 33,814 unique records. From that, we estimate it would take 40 person-months to screen all the records identified in this search, and that this would be reduced by around 29 months by the approach described here.

We have applied the algorithm to the full dataset (remaining 63,365 records) and are in the process of full-text screening. Following this process, further records will be excluded, which will allow for further training of the algorithm, to be used in future living systematic review on this topic [41].

### Error analysis

By using the ML algorithm to classify the likelihood of inclusion for each record in the training set, we highlighted discrepancies between the human and the machine decision. Using this technique, we identified human errors, which were then corrected to update the training set.

Human screening of the training set was conducted using the ‘majority vote’ system; it is interesting to consider the potential reasons for errors or ‘misclassifications’ arising in this process. Reviewers’ interpretation of the ‘breadth’ of this wide review might be one contributing factor to discrepancies; because there is not a single clearly articulated scientific question. Reviewers may be less sure which articles should be included. In smaller reviews with few contributors, it may be possible to identify some of these issues in discussion, but for larger projects using a crowd-sourcing approach with many individuals contributing decisions, this may not be a practical solution.

We have successfully identified screening errors in 1% of the training set which had been dual screened by two independent human reviewers and where disagreements had been reconciled by a third reviewer. The prevalence of inclusion in the uncorrected training set was 13.2% (760 out of the 5749), so an error of 0.8% is likely to be important. The improvement following error correction shows the impact of such errors on the learning of the ML algorithm. The error analysis results in improvement in sensitivity and specificity, with increased precision, accuracy, work saved over sampling, and positive likelihood ratio. We observed an increase in specificity of 1.6% without compromise to sensitivity. In a systematic review with this number of records, this saves considerable human resources, as the number of records required to screen reduces by over 1000.

This error analysis was an initial pilot with pragmatic stopping criteria. It is likely that there are further errors in the human screened training set. A more in-depth analysis of the training dataset, investigating every instance where the human and machine decision were incongruent, might identify more errors and further increase the precision and accuracy of machine learning approaches, further reducing human resources required for this stage of systematic review. We have shown here that even with minimal intervention (only assessing incongruent records until the original human decision was correct five consecutive times), the performance of ML approaches can be substantially improved; further improvements are likely to be less dramatic, but this is an interesting topic for future research.

### Limitations and future directions

Here, we show the best performing algorithms for this dataset with a broad research question. Other dissimilar research questions or topics may require different levels of training data to achieve the same levels of performance or may require different topic modelling approaches or classifiers. We are using the best performing algorithm described here in an ongoing research project; therefore, the ‘true’ inclusion and exclusion results for the remaining 63,365 records is not yet known.

The low precision estimate achieved by this classifier may mean it is less useful in projects where the inclusion prevalence is smaller. Where the inclusion prevalence is 5%, we calculated the precision to be 30% (Additional file 2: Figure S1, Additional file 3: Data S1). Therefore, the machine learning algorithms tested here may not be useful where the research question is a lot more specific or where systematic searches that are not very specific. One approach in cases where prevalence is low may be to adjust for a class imbalance in the training sets [42]. By manually constructing datasets and training algorithms on training sets with different ‘prevalence’ or different class imbalance, the variance in the predictions the model makes can potentially be reduced (see [43]). A complementary approach may be a refinement of the search strategy to increase the prevalence of inclusion.

These machine learning algorithms are deployed in an existing systematic review online platform, EPPI-Reviewer [28], and this functionality is in the process of being integrated into the Systematic Review Facility (SyRF) tool (app.syrf.org.uk) via an Application Programming Interface. In addition, some of these unsupervised methods have been deployed in the web-based platform RobotAnalyst [44] which combines text mining and machine learning algorithms for organising references by their context and actively prioritising them based on relevancy feedback. These functionalities will be linked to SyRF via an API.

Establishing technical standards to ensure the inter-operability of task specific automation tools with ‘whole process’ online platforms such as SyRF would allow better exploitation of new and existing tools by the wider systematic review community. Such platforms could allow individuals to select which automation tools they wished to use and to select classifiers and levels of performance appropriate to their specific research project may help integrate features.

## Conclusions

We have demonstrated that machine learning techniques can be successfully applied to an ongoing, broad pre-clinical systematic review; that they can be used to identify human errors in the training and validation datasets; and that updating the learning of the algorithm after error analysis improves performance. This error analysis technique requires further detailed elucidation and validation. These machine learning techniques are in the process of being integrated into existing systematic review applications to enable more wide-spread use. In the future, machine learning and error analysis techniques that are optimised for different types of review topics and research questions can be applied seamlessly within the existing methodological framework.

## Additional files

Additional file 1: **Table S1.** Performance of machine learning approaches on depression training dataset (1993 records). **Table S2.** Performance of machine learning approaches on depression training dataset (2989 records). (DOCX 15 kb)

Additional file 2: **Figure S1.** With the likelihood ratio of the applied algorithm after error analysis being 8.436 we can calculate the precision at different levels of prevalence of inclusion. The application of the machine learning algorithm to this systematic review which has a 14% inclusion prevalence, we can calculate the precision to be 55.9%. If the inclusion prevalence of a hypothetical review would be 5%, the precision would be approximately 30% which is poor. Therefore, the utility of applying this machine learning approach to systematic reviews with different inclusion prevalences needs to be considered. (DOCX 17 kb)

Additional file 3: Data S1. Data file to display levels of precision with different levels of prevelance of inclusion. (XLSX 17 kb)

## Abbreviations

AUC: Area under the curve
BoW: Bag-of-words
CAMARADES: Collaborative Approach to Meta-Analysis and Review of Animal Data from Experimental Studies
LDA: Latent Dirichlet allocation
LR+: Positive Likelihood Ration
LSI: Latent semantic indexing
ML: Machine learning
NRI: Net reclassification index
PROSPERO: International Prospective Register of Systematic Reviews
SGD: Stochastic gradient descent
SLIM: Systematic Living Information Machine collaboration
SVD: Singular value decomposition
SVM: Support vector machine
SYRCLE: Systematic Review Center for Laboratory animal Experimentation
SyRF: Systematic review facility
TD-IDF: Term frequency–inverse document frequency
WSS: Work saved over sampling
