# Supplementary material for: Machine learning algorithms for systematic review: reducing workload in a preclinical review of animal studies and reducing human screening error
Source: Syst Rev. 2019 Jan 15;8:23. doi: 10.1186/s13643-019-0942-7 (PMC6334440; doi:10.1186/s13643-019-0942-7)
Supplement: Supplementary file 1 — Table S1. Performance of machine learning approaches on depression training dataset (1993 records). Table S2. Performance of machine learning approaches on depression training dataset (2989 records). (DOCX 15 kb) [file 13643_2019_942_MOESM1_ESM.docx]

| **Table S1. Performance of machine learning approaches on depression training dataset (1993 records).** | | |
| --- | --- | --- |
|  | **Approach 1** | **Approach 2** |
| **Training Set Size** | **1993** | **1993** |
| **Optimal Cut-Off Score** | **0.25** | **-0.5** |
| **Sensitivity** | **99.2%** | **89.3%** |
| **Upper 95% CI** | **0.940** | **0.819** |
| **Lower 95% CI** | **0.995** | **0.939** |
| **Specificity** | **77.9%** | **89.0%** |

*Table S1 shows the performance of the two machine learning approaches trained on 1993 records.*

| **Table S2. Performance of machine learning approaches on depression training dataset (2989 records).** | |  |
| --- | --- | --- |
|  | **Approach 1** | |
| **Training Set Size** | **2989** | |
| **Optimal Cut-Off Score** | **0.25** | |
| **Sensitivity** | **96.2%** | |
| **Upper 95% CI** | **0.910** | |
| **Lower 95% CI** | **0.986** | |
| **Specificity** | **84.3%** | |

*Table S2 shows the performance of one machine learning approach trained on 2989 records.*
