# Supplementary material for: Machine learning algorithms for systematic review: reducing workload in a preclinical review of animal studies and reducing human screening error
Source: Syst Rev. 2019 Jan 15;8:23. doi: 10.1186/s13643-019-0942-7 (PMC6334440; doi:10.1186/s13643-019-0942-7)
Supplement: Supplementary file 2 — Figure S1. With the likelihood ratio of the applied algorithm after error analysis being 8.436 we can calculate the precision at different levels of prevalence of inclusion. The application of the machine learning algorithm to this systematic review which has a 14% inclusion prevalence, we can calculate the precision to be 55.9%. If the inclusion prevalence of a hypothetical review would be 5%, the precision would be approximately 30% which is poor. Therefore, the utility of applying this machine learning approach to systematic reviews with different inclusion prevalences needs to be considered. (DOCX 17 kb) [file 13643_2019_942_MOESM2_ESM.docx]

# Figure S1:

Figure S1: *With the likelihood ratio of the applied algorithm after error analysis being 8.436 we can calculate the precision at difference levels of prevalence of inclusion. The application of the machine learning algorithm to this systematic review which has a 14% inclusion prevalence, we can calculate the precision to be 55.9%. If the inclusion prevalence of a hypothetical review would be 5%, the precision would be approximately 30% which is poor. Therefore the utility of applying this machine learning approach to systematic reviews with different inclusion perveances needs to be considered.*
